# CAPAM: The mRNA Cap Adenosine *N*6-Methyltransferase

**DOI:** 10.1016/j.tibs.2019.01.002

**Published:** 2019-03

**Authors:** Victoria H. Cowling

**Affiliations:** 1 Centre for Gene Regulation and Expression, School of Life Sciences, University of Dundee, Dow Street, Dundee DD1 5EH, UK

**Keywords:** m6Am, mRNA cap, mRNA cap methyltransferase, CAPAM, Pcif1, gene expression

## Abstract

The mRNA cap is a structure that protects mRNA from degradation and recruits processing and translation factors. A new mRNA capping enzyme has been identified, PCIF1/CAPAM, which methylates adenosine when it is the first transcribed nucleotide. This discovery is crucial for understanding the function of cap adenosine methylation.

During eukaryotic gene expression, pre-mRNA is modified, spliced, and exported into the cytoplasm where it is translated into protein. Because mRNA constitutes a small proportion of cellular RNA it requires a mark of identity – a methylated structure at the 5′ terminus called the mRNA cap – to be selected for mRNA-specific processing and translation 1, 2. The cap structure varies in different species: in mammals the predominant form is denoted m7G(5′)ppp(5′)Xm, in which 7-methylguanosine (m7G) is linked to the first transcribed nucleotide (X) via a 5′ to 5′ triphosphate bridge, and the first transcribed nucleotide is methylated (m) at the ribose *O*2 position. If the first transcribed nucleotide is adenosine it can be further methylated on the *N*6 position. The mRNA cap specifically forms on the first nucleotides of RNA transcribed by RNA polymerase II (Pol II), including pre-mRNA, because the capping enzymes are recruited to phospho-RNA Pol II during transcription (Figure 1). The mRNA cap protects pre-mRNA from nucleases, recruits cap-binding proteins involved in RNA processing and translation initiation, and protects mRNA from attack by the innate immune response. A novel cap methyltransferase, PCIF1/CAPAM, was recently identified 3, 4, 5, 6. These findings and their implications for gene expression control are discussed here.

**Figure 1:**
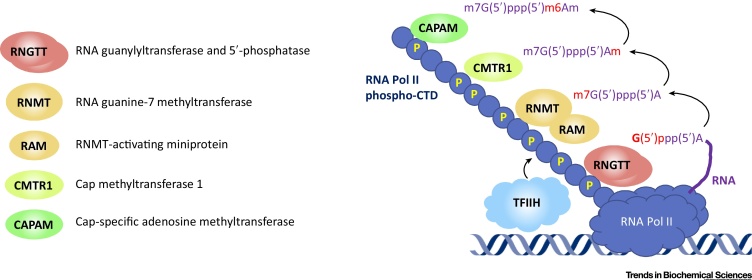
mRNA Capping Enzymes Recruited to RNA Polymerase II (Pol II) During Transcription. During the initial phase of transcription, TFIIH phosphorylates the Pol II C-terminal domain (CTD) on serine 5. The capping enzymes RNGTT, RNMT–RAM, CMTR1, and CAPAM are then recruited to the phosphorylated RNA Pol II. Next to each capping enzyme is a cap structure that they produce, with the specific component added marked in red. RNA is synthesised with a 5′ triphosphate. RNGTT is a triphosphatase and guanylyltransferase that adds the inverted guanosine cap to nascent RNA. The other enzymes, RNMT-RAM, CMTR1 and CAPAM, are methyltransferases. RNA is depicted as a purple strand.

The discovery of the mRNA cap began in viruses and mammalian cells using mass spectrometry and biochemistry [1]. The crucial role of the cap in mRNA stability, processing, and translation was initially revealed using *in vitro* assays. However, to understand the role of the different cap modifications it is essential to identify the enzymes involved. This allows the capping enzyme and the pre-mRNA modification it catalyses to be ablated in cells, and the impact on gene expression and cell function to be determined. Because many RNA processing events are mechanistically linked to the processes which occur before and after (transcription and capping, capping and splicing, etc.), it is important to analyse RNA processing mechanisms in intact cells. The enzymes which initiate cap formation are present in all eukaryotes and have been most extensively investigated in yeast species. However, some cap modifications, including methylation of the first transcribed nucleotide, are restricted to higher eukaryotes, and perhaps as a result the enzymes responsible have been elusive.

Recently a novel cap methyltransferase was discovered; CAPAM (cap-specific adenosine *N*6-methyltransferase), which catalyses *N*6-methylation of the first transcribed nucleotide adenosine to create the cap structure m7G(5′)ppp(5′)m6Am 3, 4, 5, 6 (Figure 1). m7G(5′)ppp(5′)m6Am is an abundant cap, and therefore has the potential to be biologically important. In HEK293T cells, 92% of mRNA initiating with adenosine has a m7G(5′)ppp(5′)m6Am cap, although this can fluctuate in different cell lineages 3, 4, 5, 6, 7. Furthermore, mRNAs starting with m7G(5′)ppp(5′)m6Am are on average more stable and highly expressed than mRNAs with other caps [4]. CAPAM was previously identified as PCIF1 [human phosphorylated C-terminal domain (CTD)-interacting factor 1], and was found to negatively impact on RNA Pol II-dependent transcription [8]. All recent studies agree that CAPAM is the only cap-specific adenosine *N*6-methyltransferase 3, 4, 5, 6. Furthermore, CAPAM does not methylate adenosine residues in the RNA body 3, 4, 5, 6.

On comparing the structures of CAPAM to the other mammalian cap methyltransferases (RNMT, CMTR1, CMTR2), the catalytic domains are observed to have homology; however, the surrounding regions vary, indicating different mechanisms of action and regulation 2, 3. CAPAM has a catalytic subunit containing a methyltransferase domain which has a canonical Rossmann fold with a conserved catalytic motif and a ‘helical domain’ consisting of multiple helices and β-sheets [3]. The helical domain is intriguing because it does not have overt homology to other solved structures. As with the first nucleotide ribose *O*2-methyltransferase, CMTR1, CAPAM has a WW domain through which it interacts with the serine-5 phosphorylated CTD of RNA Pol II 2, 3 (Figure 1).

The identification of CAPAM as the cap adenosine *N*6-methyltransferase has begun to reveal important facets of m7G(5′)ppp(5′)m6A cap function. Akichika *et al*. noted little impact of CAPAM knockout on cell proliferation under normal tissue culture conditions [3]. However, a significant proliferative defect was observed under conditions of oxidative stress, and it may be that CAPAM has a prominent biological role in specialised cell functions or in specific cell lineages. Previously m7G(5′)ppp(5′)m6Am had been found to stabilise transcripts [9]. Following CAPAM knockout, Akichika *et al*. observed slight increases and decreases in steady-state mRNA levels. mRNAs starting with an adenosine were increased with respect to other mRNAs, suggesting that CAPAM or m7G(5′)ppp(5′)m6Am represses these transcripts (decreasing transcription or RNA stability) [3]. Sendinc *et al*. also reported increases and decreases in mRNA expression on ablation of CAPAM, and these correlated with changes in transcription rather than in RNA stability [5]. These CAPAM-dependent changes in transcription were independent of m7G(5′)ppp(5′)m6Am, suggesting either indirect effects of adenosine *N*6-methylation or methyltransferase-independent impacts of CAPAM. Of note, the guanosine cap *N*7-methyltransferase, RNMT–RAM, has methyltransferase-independent effects on transcription [10]. Because CAPAM binds to RNA Pol II, it may also have methyltransferase-independent effects on transcription. Indeed, in 2008 Hirose *et al*. reported that PCIF1 negatively regulates transcription, although the mechanism remains unclear [8]. Boulias *et al*. [4] noted that changes in mRNA expression following CAPAM knockout depended on the basal mRNA expression level. High-abundance, stable m7G(5′)ppp(5′)m6Am-capped mRNAs did not significantly change in expression following CAPAM knockout, whereas low-abundance and less-stable m7G(5′)ppp(5′)m6Am-capped mRNAs were reduced. Although these studies appear somewhat contradictory in detail, CAPAM clearly has direct and indirect impacts on RNA stability and transcription, the net output of which may depend on subtle changes in cell physiology.

The impact of CAPAM on translation was also investigated. Akichika *et al*. observed that CAPAM knockout decreased the translation of a subset of mRNAs which are enriched for the m7G(5′)ppp(5′)m6Am cap [3]. The fact that not all translationally repressed mRNAs contain the m7G(5′)ppp(5′)m6Am cap again suggests direct and indirect impacts of CAPAM. In other studies using reporter constructs and RNAs, the presence of the m7G(5′)ppp(5′)m6Am cap had the opposite or minimal effect on translation 5, 9. Thus the m7G(5′)ppp(5′)m6Am may have gene- and cell-specific impacts. Sendinc *et al*. report that first-nucleotide adenosine methylation decreases binding to eIF4E, a cap-binding protein that promotes ribosomal subunit recruitment [5]. The list of cap-binding proteins which impact on translation is continually increasing, and it may be that cap adenosine methylation alters the relative affinity for these proteins, which could explain the observed differential effects of CAPAM on translation 2, 3, 5.

In summary, the identification of CAPAM as the first-nucleotide adenosine *N*6-methyltransferase is a major finding which will allow the biological function of this modification to be uncovered. Once the physiological processes in which CAPAM has an influential role are identified, the impact of this enzyme and the m7G(5′)ppp(5′)m6Am cap on gene expression may be clarified.

## References

[1] Furuichi Y. (2015). Discovery of m^7^G-cap in eukaryotic mRNAs. Proc. Jpn. Acad. B Phys. Biol. Sci..

[2] Galloway A., Cowling V.H. (2018). mRNA cap regulation in mammalian cell function and fate. Biochim. Biophys. Acta Gene Regul. Mech..

[3] Akichika S. (2019). Cap-specific terminal N^6^-methylation of RNA by an RNA polymerase II-associated methyltransferase. Science.

[4] Boulias K. (2018). Identification of the m6Am methyltransferase PCIF1 reveals the location and functions of m6Am in the transcriptome. bioRxiv.

[5] Sendinc E. (2018). PCIF1 catalyzes m6Am mRNA methylation to regulate gene expression. bioRxiv.

[6] Sun H. (2019). Cap-specific, terminal N^6^-methylation by a mammalian m^6^Am methyltransferase. Cell Res..

[7] Kruse S. (2011). A novel synthesis and detection method for cap-associated adenosine modifications in mouse mRNA. Sci. Rep..

[8] Hirose Y. (2008). Human phosphorylated CTD-interacting protein, PCIF1, negatively modulates gene expression by RNA polymerase II. Biochem. Biophys. Res. Commun..

[9] Mauer J. (2017). Reversible methylation of m6Am in the 5′ cap controls mRNA stability. Nature.

[10] Varshney D. (2018). mRNA cap methyltransferase, RNMT–RAM, promotes RNA Pol II-dependent transcription. Cell Rep..

